# Regadenoson and adenosine are equivalent vasodilators and are superior than dipyridamole- a study of first pass quantitative perfusion cardiovascular magnetic resonance

**DOI:** 10.1186/1532-429X-15-85

**Published:** 2013-09-24

**Authors:** Sujethra Vasu, W Patricia Bandettini, Li-Yueh Hsu, Peter Kellman, Steve Leung, Christine Mancini, Sujata M Shanbhag, Joel Wilson, Oscar Julian Booker, Andrew E Arai

**Affiliations:** 1 Section of Cardiology, Wake Forest University School of Medicine, Medical Center Blvd, Winston-Salem, NC, USA; 2 Cardiovascular and Pulmonary Branch, National Heart, Lung and Blood Institute, National Institutes of Health, Department of Health and Human Services, 10 Center Drive, Building 10, Room B1D416, 20892-1061 Bethesda, MD, USA; 3 Department of Cardiology, University of Alabama, Birmingham, AL, USA

**Keywords:** Regadenoson, Dipyridamole, Adenosine, Quantitative myocardial perfusion, Magnetic resonance imaging

## Abstract

**Background:**

Regadenoson, dipyridamole and adenosine are commonly used vasodilators in myocardial perfusion imaging for the detection of obstructive coronary artery disease. There are few comparative studies of the vasodilator properties of regadenoson, adenosine and dipyridamole in humans. The specific aim of this study was to determine the relative potency of these three vasodilators by quantifying stress and rest myocardial perfusion in humans using cardiovascular magnetic resonance (CMR).

**Methods:**

Fifteen healthy normal volunteers, with Framingham score less than 1% underwent vasodilator stress testing with regadenoson (400 μg bolus), dipyridamole (0.56 mg/kg) and adenosine (140 μg /kg/min) on separate days. Rest perfusion imaging was performed initially. Twenty minutes later, stress imaging was performed at peak vasodilation, i.e. 70 seconds after regadenoson, 4 minutes after dipyridamole infusion and between 3–4 minutes of the adenosine infusion. Myocardial blood flow (MBF) in ml/min/g and myocardial perfusion reserve (MPR) were quantified using a fully quantitative model constrained deconvolution.

**Results:**

Regadenoson produced higher stress MBF than dipyridamole and adenosine (3.58 ± 0.58 vs. 2.81 ± 0.67 vs. 2.78 ± 0.61 ml/min/g, p = 0.0009 and p = 0.0008 respectively). Regadenoson had a much higher heart rate response than adenosine and dipyridamole respectively (95 ± 11 vs. 76 ± 13 vs. 86 ± 12 beats/ minute) When stress MBF was adjusted for heart rate, there were no differences between regadenoson and adenosine (37.8 ± 6 vs. 36.6 ± 4 μl/sec/g, p = NS), but differences between regadenoson and dipyridamole persisted (37.8 ± 6 vs. 32.6 ± 5 μl/sec/g, p = 0.03). The unadjusted MPR was higher with regadenoson (3.11 ± 0.63) when compared with adenosine (2.7 ± 0.61, p = 0.02) and when compared with dipyridamole (2.61 ± 0.57, p = 0.04). Similar to stress MBF, these differences in MPR between regadenoson and adenosine were abolished when adjusted for heart rate (2.04 ± 0.34 vs. 2.12 ± 0.27, p = NS), but persisted between regadenoson and dipyridamole (2.04 ± 0.34 vs. 1.77 ± 0.33, p = 0.07) and between adenosine and dipyridamole (2.12 ± 0.27 vs. 1.77 ± 0.33, p = 0.01).

**Conclusions:**

Based on fully quantitative perfusion using CMR, regadenoson and adenosine have similar vasodilator efficacy and are superior to dipyridamole.

## Background

Regadenoson, dipyridamole and adenosine are commonly used, FDA approved vasodilators for the noninvasive detection of obstructive coronary artery disease (CAD) using myocardial perfusion imaging [1,2]. Regadenoson is a newer, selective adenosine 2A receptor agonist initially studied in myocardial perfusion imaging with nuclear scintigraphy techniques [3,4]. Regadenoson is a more potent vasodilator than adenosine and exhibits selectivity for the coronary circulation relative to the renal, peripheral and mesenteric circulation in animals [5]. Adenosine is nonselective and causes negative chronotropic, dromotropic and inotropic effects via A1 receptors. It also causes bronchospasm and mast cell degranulation via A3 receptors. In contrast, dipyridamole induces vasodilation indirectly, by blocking adenosine reuptake and increasing endogenous adenosine.

In animal studies, regadenoson was a more potent vasodilator than adenosine. The higher median effective dose, defined as the dose producing 50% of maximum effect was 0.34 ± 0.08 μg/kg for regadenoson and 51 ± 15 μg/kg for adenosine. The increase in CBF with regadenoson reached 84 ± 5% of maximal reactive hyperemia. Both vasodilators had similar maximal increase in coronary blood flow (CBF) [6] and produced similar hemodynamic effects and biodistribution of radiotracers as assessed with nuclear perfusion imaging [7].

The hemodynamic effects of regadenoson have been studied in humans by measuring the increase in the peak coronary flow velocity (CFV) using intracoronary pulsed Doppler. This dose ranging study, using intravenous administration of 10-500 μg of regadenoson assessed the increase in the average peak CFV in 34 human subjects. The mean peak increases in CFV ± SD with intravenous regadenoson at doses of 10, 30, 100, 300, 400, and 500 μg were 1.8 ± 0.57, 2.5 ± 0.54, 3.0 ± 0.61, 3.4 ± 0.77, 3.1 ± 0.52, and 3.1 ± 0.79 times respectively higher than baseline [8]. A dose of 400 μg regadenoson was not inferior compared to adenosine in the detection of the extent and severity of perfusion defects [9]. However, comparative studies of quantitative perfusion with regadenoson, dipyridamole and adenosine have not been performed in humans.

First pass perfusion using CMR is accurate in the detection of coronary artery disease [10-15] and in the quantification of MBF [16-19]. Until recently, dipyridamole and adenosine have been commonly used in CMR perfusion imaging protocols. Regadenoson has desirable factors such as bolus administration that can simplify stress perfusion CMR.

The specific aim of this study was to compare the effectiveness of regadenoson vs. dipyridamole vs. adenosine using first pass quantitative perfusion CMR in healthy normal volunteers. We hypothesized that, if regadenoson causes a significantly higher increase in the stress MBF compared to adenosine or dipyridamole, then quantitative perfusion CMR will be able to demonstrate differences between these agents.

## Methods

### Study population

The inclusion criteria for volunteers were: absence of chest pain for the past 6 months, no smoking history, and no cardiac risk factors. Exclusion criteria were standard CMR contradictions such as cerebral aneurysm clips, metal in the eye, and implanted metallic devices.

### Study design

All volunteers were asked to abstain from caffeinated products for at least 24 hours. Caffeine levels were drawn on the day of each CMR scan to document compliance.

**Figure 1:**
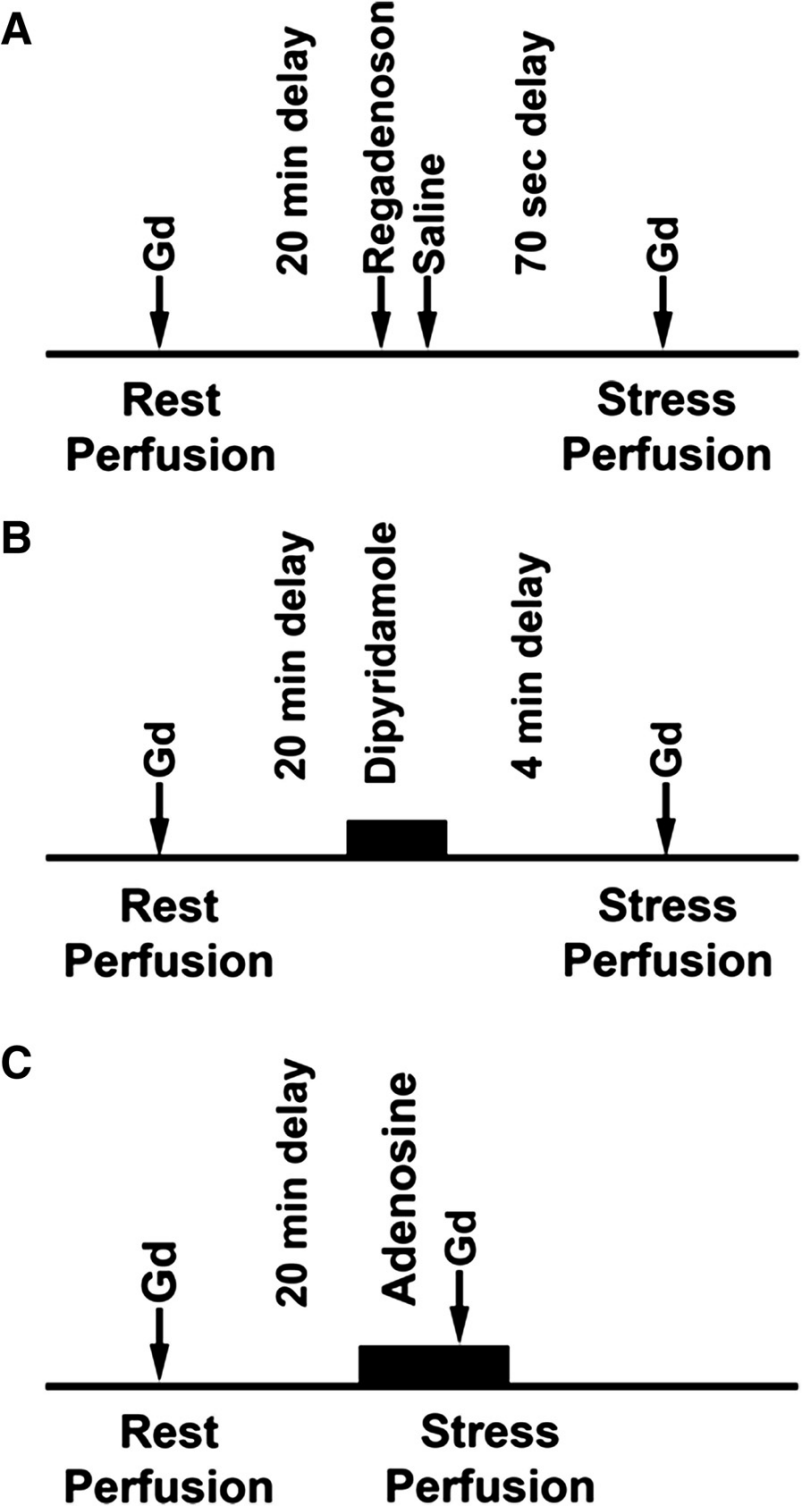
**A, B and C: study design using regadenoson, dipyridamole and adenosine.** Sixteen volunteers underwent stress perfusion studies in three separate exams using regadenoson, dipyridamole and adenosine. Gadolinium contrast is shown as Gd. Minutes and seconds are shown as min and sec respectively.

The study design is shown below (Figure 1A, B and C). Rest imaging was performed first followed by stress imaging 20 minutes later. Regadenoson was administered as a 400 mcg dose over 10 seconds, followed by a 10 ml saline flush. Dipyridamole was given as a 0.56 mg/kg infusion for 4 minutes and adenosine as a 140 mcg/kg infusion for 5–6 minutes. Stress imaging was performed at peak vasodilation defined as 70 seconds after regadenoson bolus, 4 minutes after dipyridamole infusion, and 3–4 minutes after the start of the adenosine infusion. This study had institutional review board approval and all subjects provided written informed consent.

The order of studies with a specific vasodilator was not randomized. All volunteers underwent stress testing with regadenoson, followed by dipyridamole and then adenosine on different days. All studies were performed between 8 am and noon. The average duration between the regadenoson and dipyridamole scans was 18 days and the duration between dipyridamole and the adenosine scans was 67 days. Five of the original volunteers returned for a fourth visit for interstudy reproducibility with regadenoson. The average duration between the first scan with regadenoson and the repeat scan was 54 days.

### CMR protocol

All volunteers were scanned with a 1.5 T Siemens scanner (Espree, Siemens Medical Solutions, Erlangen, Germany). Rest and stress imaging was performed, each with 0.05 mmol/kg of gadolinium-DTPA (Magnevist, Berlex Laboratories, Wayne, NJ, USA), diluted to provide injections of equal volumes and flushed with saline at 5 mL/sec flow rate (Medrad, Indianola, PA, USA).

A saturation recovery-prepared steady state free precession sequence was used to acquire three slice locations (base, mid, and apex) every R-R interval for a period lasting 60 heartbeats. Typical imaging parameters included a saturation preparation pulse, readout excitation flip angle 50°, repetition time/ echo time 2.3/ 1.1 ms, bandwidth 1085 Hz/pixel, acquisition matrix 128 × 80, field of view 360 × 270 mm, slice thickness 8 mm, with parallel imaging acceleration factor of 2. The acquisition shot duration was 92 ms. A separate saturation preparation pulse was used for each slice. The time per slice was 132 ms which included the saturation preparation pulse, delay and imaging. A proton density-weighted reference image was acquired to facilitate surface coil intensity correction, at the beginning of perfusion imaging, using a small magnetization flip angle (8°) and no saturation preparation pulse. For the arterial input function (AIF) acquisition, only the mid slice was acquired every RR interval. A separate saturation prepared, low resolution image with FLASH readout, acquisition matrix 48 × 64, temporal resolution 60 ms was acquired at the beginning of each RR interval for arterial input function (AIF) assessment as described by Gatehouse et al [20]. This was followed by the myocardial perfusion imaging whose parameters have been described above.

### Myocardial blood flow quantification

In healthy normal volunteers, the stress MBF is not expected to have regional differences among the basal, mid and apical segments. The apical slices were not analyzed. Only the basal and mid slices were analyzed with similar estimates of MBF. To conform to the commonly reported mid-ventricular slice in studies of quantitative myocardial perfusion, we have reported the results from the mid ventricular slice only. The mid myocardial perfusion slice was divided into six radial sectors. Contours of the left ventricular (LV) epicardial and endocardial borders were manually traced on each image. Time-signal intensity curves of myocardial regions of interest (ROIs) were generated and analyzed using custom software written in Interactive Data Language (Research Systems Inc Boulder, CO, USA). The AIF was quantified by drawing ROIs in the LV cavity in the low resolution images acquired concurrently with myocardial perfusion images. In first-pass perfusion imaging, the time-signal intensity measurements within the heart reflect the contrast concentration during the wash-in and wash-out of the bolus.

We used a modified Fermi function deconvolution method to quantify MBF in ml/min/g and MPR as previously described by Hsu et al [17], except the AIF was imaged using a dual sequence method as described by Gatehouse et al [20]. MBF in each of the six sectors were fit individually from the ROIs and then averaged for every volunteer. None of the ROIs were discarded due to poor fits.

The image analysts were not blinded to the stress agent used. There were multiple steps in the workflow including image processing for myocardial perfusion and AIF assessment, and the MBF analysis which were done in separate time periods.

### Statistical analysis

Data was analyzed using MedCalc version. 11.6. All data are presented as mean ± standard deviation. A sample size of 15 in each group of normal volunteers was required to detect a 25% increase in resting MBF with regadenoson, with an alpha of 0.05 and a power of 0.8. Our first criterion for a better vasodilator was a higher stress MBF. The next criterion was to observe an increase in stress MBF on a per subject basis. Inter-observer, intra-observer reproducibility and repeatability between two studies were analyzed in 5 subjects using Bland-Altman plots. In addition the coefficient of variation was also used to assess reproducibility.

Differences in absolute myocardial blood flow between the three vasodilators were assessed using ANOVA with repeated measures and with Bonferroni correction for multiple comparisons. In addition, the myocardial blood flow was adjusted for heart rate and compared between the three drugs. Correlation between the heart rate and stress myocardial blood flow was assessed using Spearman’s correlation coefficient.

## Results

The rest and stress MBF for each of the three vasodilators followed a normal distribution as confirmed with the Kolmogorov-Smirnov test of normality.

**Figure 2:**
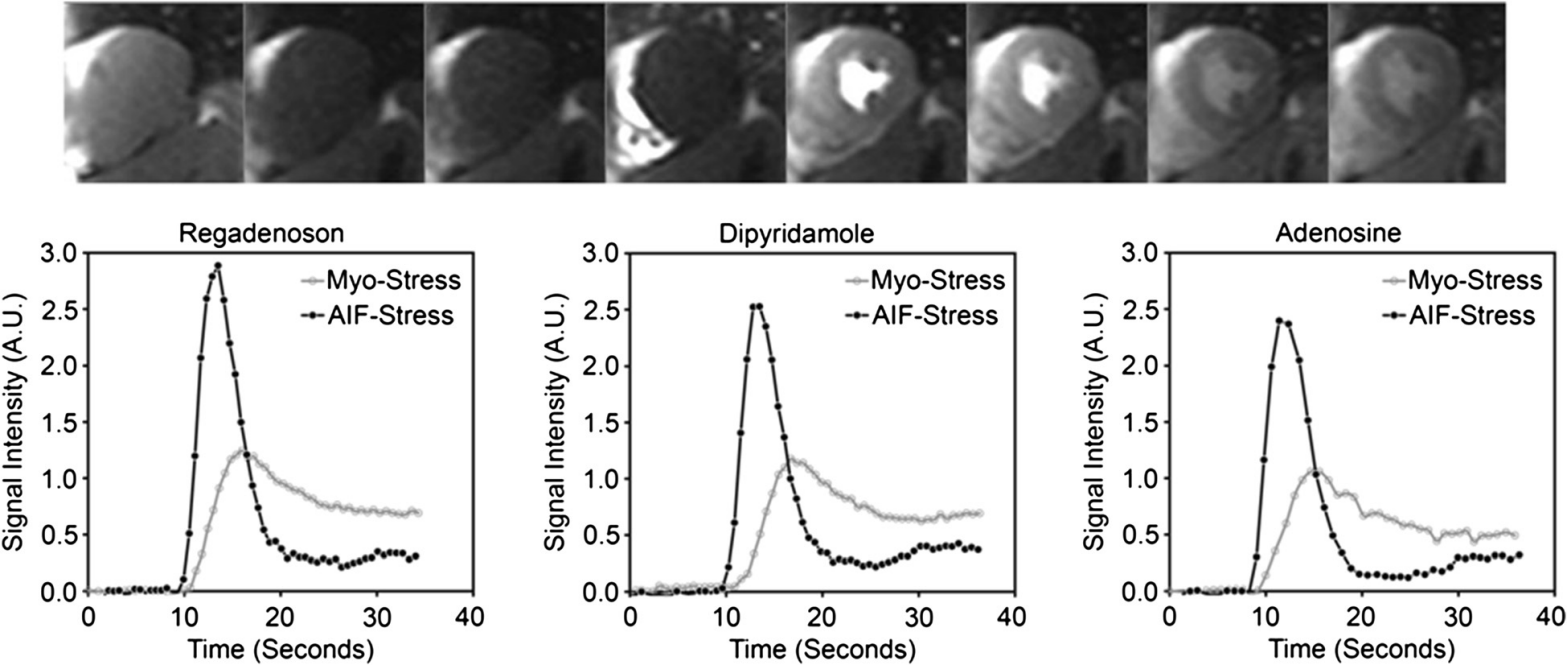
**First pass perfusion imaging and analysis of signal intensity.** The upper row shows still frame images of the first pass perfusion. The first image is a proton density weighted image. The subsequent images show the transit of contrast through the right ventricle, left ventricle and subsequently myocardial perfusion. ROIs can be used to measure signal intensity as a function of time to quantitatively analyze perfusion. The lower row shows time intensity curves of a single volunteer with regadenoson, dipyridamole and adenosine. The time intensity curves of the myocardium and the arterial input function during stress are shown as Myo-Stress and AIF-Stress respectively. Signal intensity is displayed as arbitrary units (A.U).

The 16 healthy volunteers had a Framingham score less than 1% by design and thus a very low risk profile with respect to CAD as summarized in Table 1. None of these subjects reported any prior history of chest pain that would have warranted medical evaluation. Figure 2 shows typical time-signal intensity curves of a first-pass perfusion CMR study using regadenoson, dipyridamole and adenosine. All of the volunteers, except one had undetectable caffeine levels. This volunteer had a caffeine level of 1.1 μg/ml on the day of adenosine stress imaging and was excluded from the analysis. The final sample size was 15 healthy volunteers.

**Table 1:** Demographics of the study population

**Characteristics**	**Mean ± SD or percentage**
Age (median, interquartile range)	21, 3.5
Gender	
Male	94%
Female	6%
Body mass index (kg/m2 ± SD)	24.7 ± 2.8
Smoking	0%
Hypertension	0%
Diabetes	0%
Hyperlipidemia	0%
Total- Cholesterol (mg/dl ± SD)	147 ± 38
LDL- Chol (mg/dl ± SD)	86 ± 35
HDL-Chol (mg/dl ± SD)	49 ± 17
Triglyceride (mg/dl ± SD)	62 ± 30

*SD* is defined as standard deviation, *mg/dl* as milligram/deciliter.

**Figure 3:**
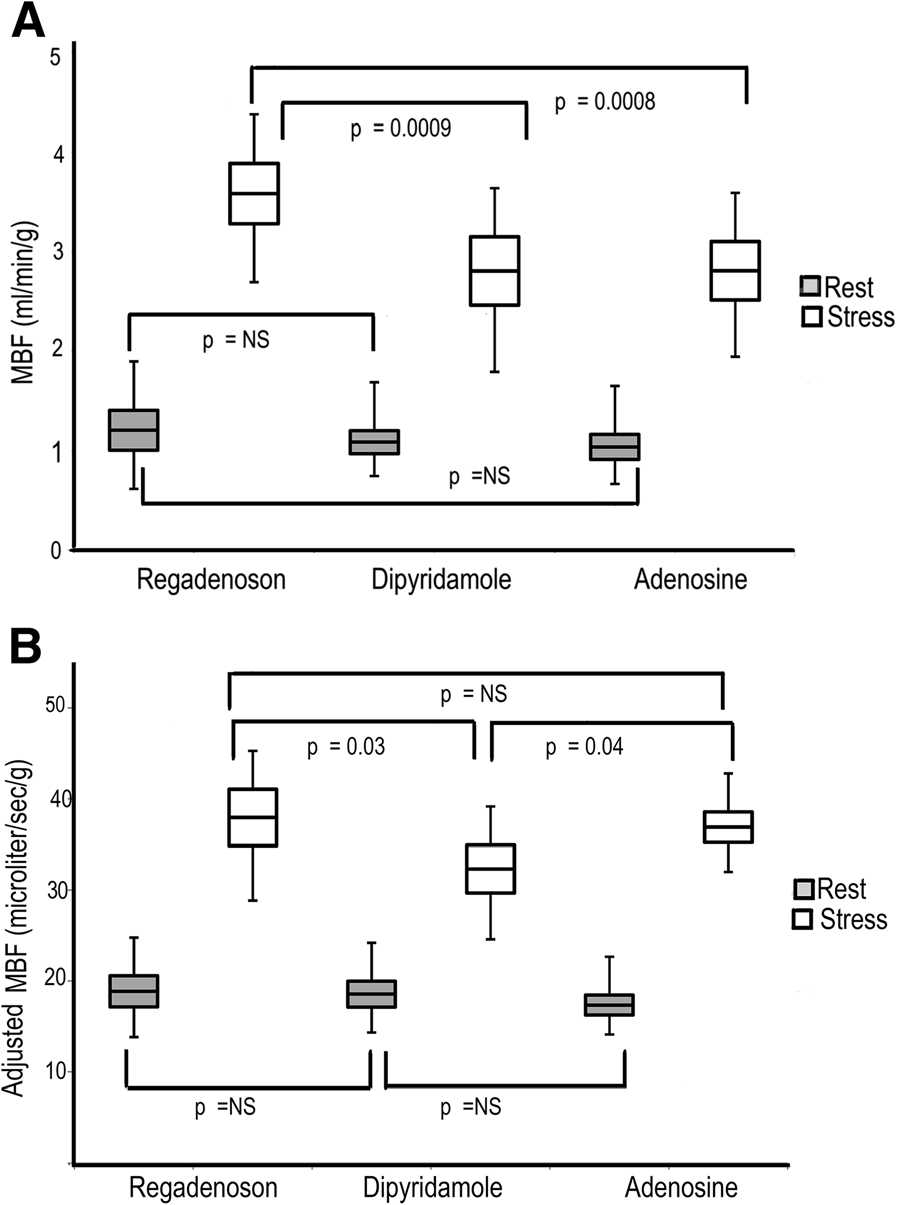
**Unadjusted and adjusted rest and stress MBF with all three stress agents in 3A and 3B respectively.** Regadenoson has a statistically significant higher unadjusted stress MBF than dipyridamole and adenosine. No difference in the unadjusted stress MBF between dipyridamole and adenosine. No difference in the resting MBF between all three agents is noted. However after adjusting for heart rate, both regadenoson and adenosine have similar stress MBF, which is higher than that of dipyridamole. Data in the box plot are represented as mean ± standard error and the whiskers represent the standard deviation.

Stress MBF (ml/min/g) was significantly higher for regadenoson, 3.58 ± 0.58 when compared to dipyridamole, 2.81 ± 0.67, p = 0.0009, and adenosine, 2.78 ± 0.61, p = 0.0008 (Figure 3A). No statistically significant difference in resting MBF between the three vasodilators was noted (regadenoson, 1.21 ± 0.38, dipyridamole, 1.09 ± 0.22 and adenosine 1.04 ± 0.24, p = NS).

When stress MBF was adjusted for heart rate, there were no differences between regadenoson and adenosine (37.8 ± 6 vs. 36.6 ± 4 μl/sec/g, p = NS), but differences between regadenoson and dipyridamole persisted. (37.8 ± 6 vs. 32.6 ± 5 μl/sec/g, p = 0.03) as shown in Figure 3B. Adenosine had higher MBF (36.6 ± 4 vs. 32.6 ± 5 μl/sec/g, p = 0.04) after adjusting for heart rate as shown in Figure 3B. This confirms that the heart rate is a major driver of the higher MBF response with regadenoson.

**Figure 4:**
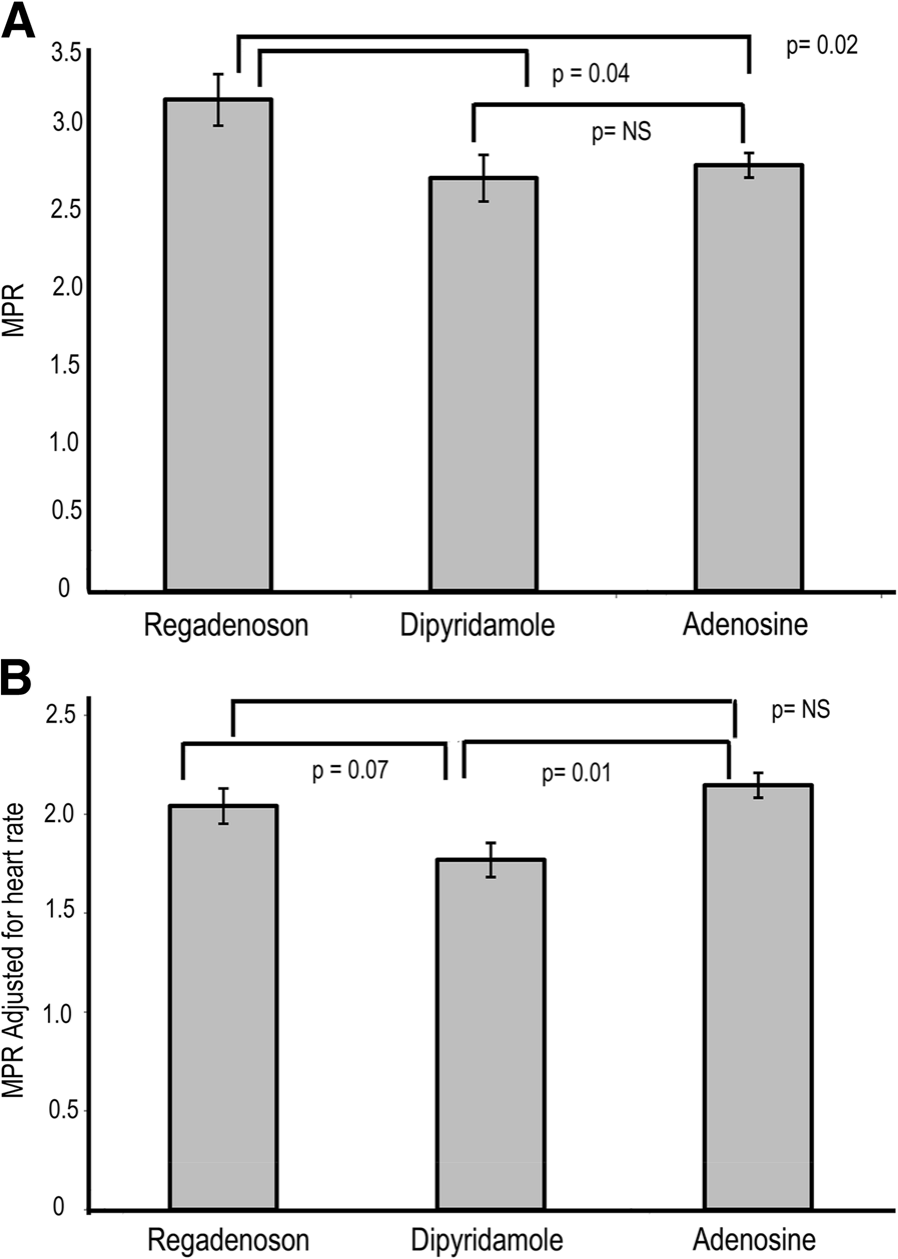
**Unadjusted and adjusted MPR with all three stress agents in 4A and 4B respectively.** Regadenoson has a statistically significant higher unadjusted stress MPR than dipyridamole and adenosine. However after adjusting for heart rate, both regadenoson and adenosine have similar MPR, which is higher than that of dipyridamole.

MPR was significantly higher with regadenoson than dipyridamole (3.11 ± 0.62 vs. 2.61 ± 0.57, p = 0.04) and adenosine (3.11 ± 0.62 vs. 2.7 ± 0.30, p = 0.02), as shown in Figure 4A. No significant differences in rest, stress MBF and the MPR was noted between dipyridamole and adenosine.

Similar to stress MBF, the differences in MPR between regadenoson and adenosine were abolished when adjusted for heart rate (2.04 ± 0.34 vs. 2.12 ± 0.27, p = NS). However differences between regadenoson and dipyridamole persisted with a trend towards statistical significance (2.04 ± 0.34 vs. 1.77 ± 0.33, p = 0.07) as shown in Figure 4B. Adenosine had higher MPR than dipyridamole (2.12 ± 0.27 vs. 1.77 ± 0.33, p = 0.01) after adjusting for heart rate as shown in Figure 4B.

**Figure 5:**
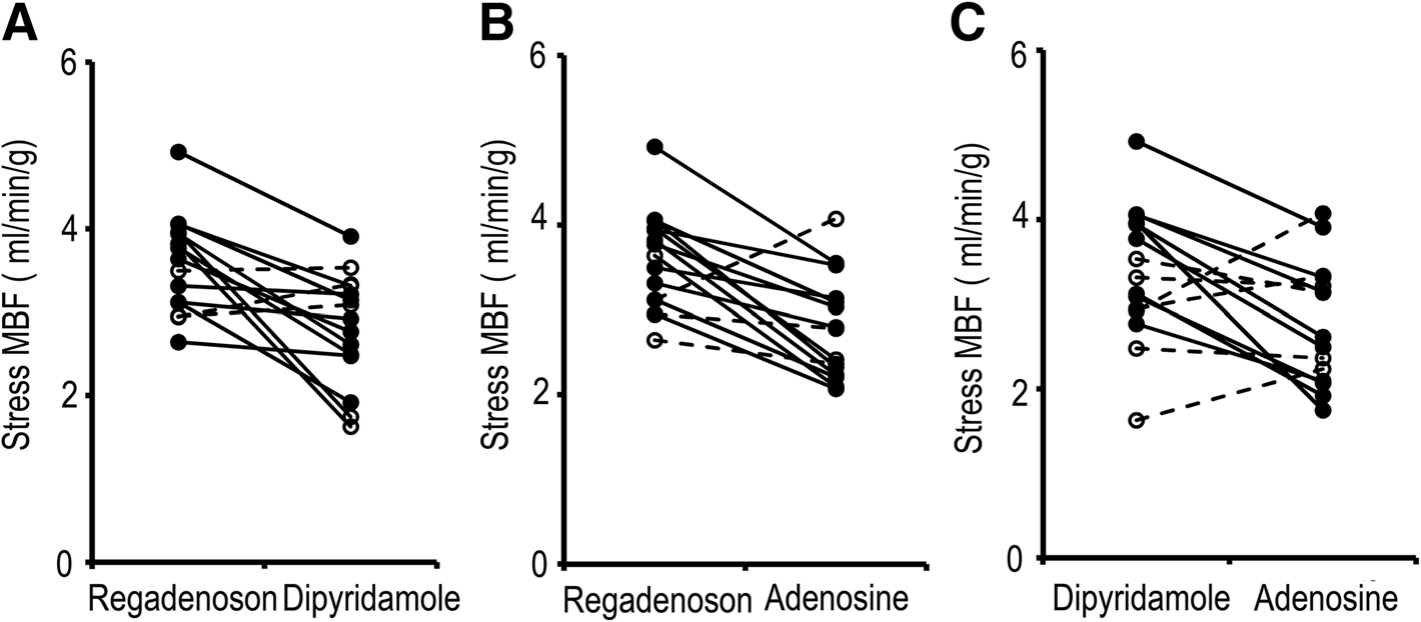
**Stress MBF on a per-subject basis between two drugs in 5A, B and C.** Stress MBF with regadenoson and dipyridamole is noted in **5A**. The 12/15subjects with a lower stress MBF response to dipyridamole when compared to regadenoson are shown in solid lines. Stress MBF with regadenoson and adenosine is noted in **5B**. The 11/15 subjects with a lower stress MBF response to adenosine when compared to regadenoson are shown in solid lines. Stress MBF with dipyridamole and adenosine is noted **5C**. 9/ 15 subjects had a higher MBF with dipyridamole than with adenosine.

The individual stress MBF with regadenoson, dipyridamole and adenosine on a per-subject basis is noted in Figure 5. While there is a wide range of absolute blood flow, 12 of 15 subjects had higher stress MBF with regadenoson compared to adenosine as shown in Figure 5A. Similarly, 11 of 15 subjects had higher stress MBF with regadenoson compared to dipyridamole as shown in Figure 5B. When dipyridamole was compared with adenosine, 9 of 15 had higher MBFs with dipyridamole as shown in Figure 5C.

**Figure 6:**
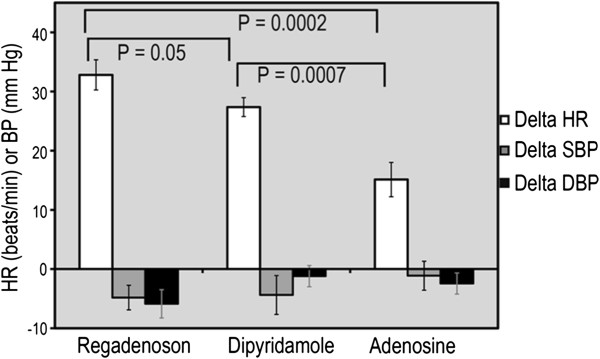
**Hemodynamic changes with regadenoson, dipyridamole and adenosine.** Compared to dipyridamole and adenosine, regadenoson induced a larger increase in heart rate (HR) with no difference in systolic or diastolic blood pressure (SBP, DBP) reduction.

There were no differences in the resting heart rate between each of the three vasodilators (63 ± 12 vs. 59 ± 8 vs. 60 ± 9) for regadenoson, dipyridamole and adenosine respectively. Regadenoson had a much higher heart rate response than adenosine (95 ± 11 vs. 76 ± 13 beats/minute) and dipyridamole (95 ± 11 vs. 86 ± 12 beats/minute). The greatest increase in heart rate was noted with regadenoson (31 ± 2.5 beats per minute), compared with dipyridamole (27 ± 6, p = 0.05) and adenosine (16 ± 8, p = 0.0002) as shown in Figure 6. Dipyridamole had a statistically higher increase in heart rate when compared to adenosine (p < 0.001). Mean change in systolic blood pressure (SBP) was −4.8 ± 9 mmHg with regadenoson, -4.4 ± 13 mmHg with dipyridamole and −0.7 ± 10 mmHg with adenosine. Mean change in diastolic blood pressure was −5.9 ± 9 mm Hg with regadenoson, -0.9 ± 7 mmHg with dipyridamole and −2.4 ± 7 mm Hg with adenosine. No statistically significant differences in blood pressure changes were found between these three vasodilators.

**Figure 7:**
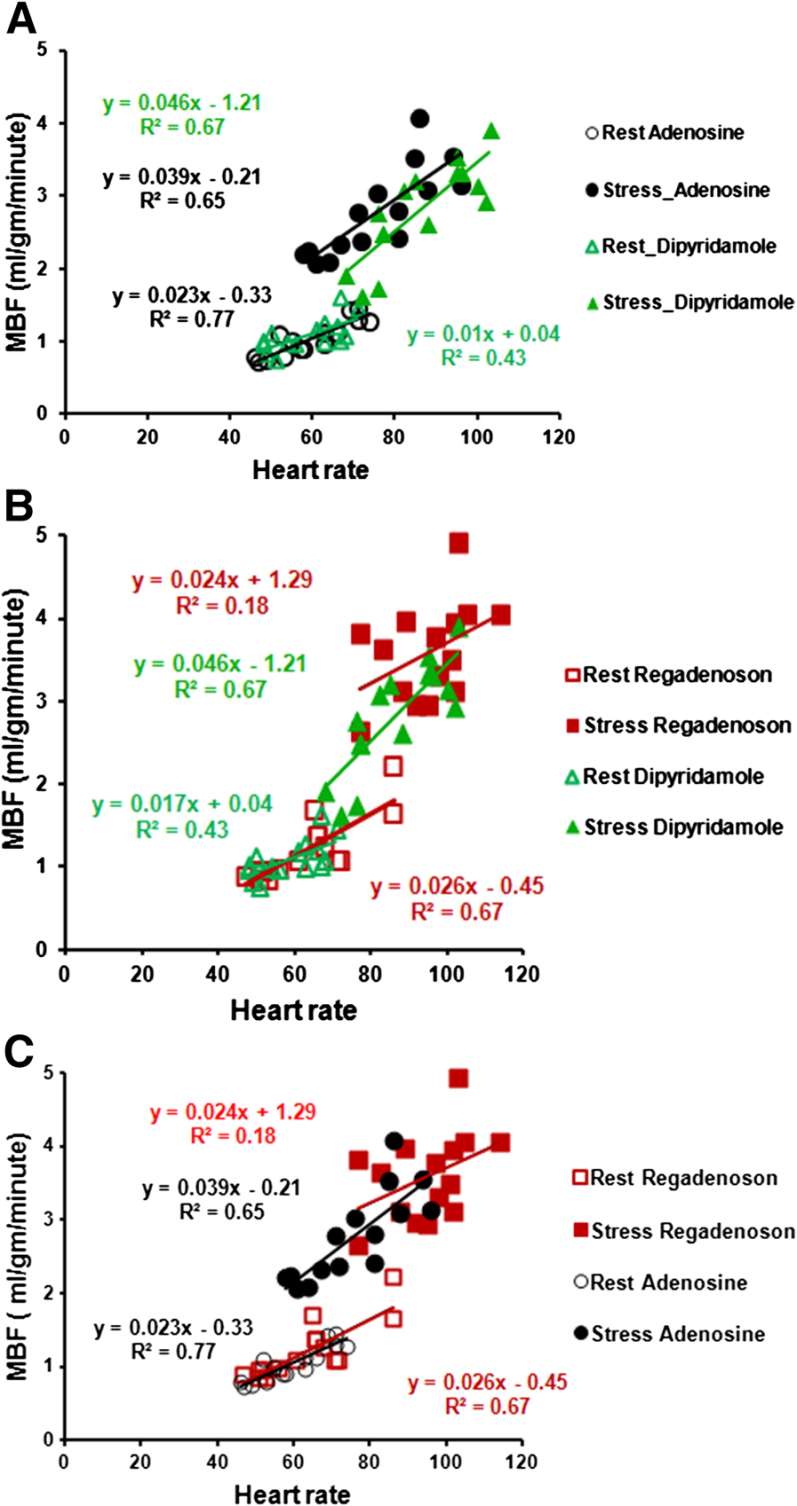
**A, B and C: correlation between heart rate and MBF is shown in a two-drug comparison.** As shown in **7A**, adenosine has a parallel upward shift ie. a higher MBF for the same heart rate, compared to dipyridamole. In contrast, regadenoson has an upward and rightward shift i.e. higher MBF and heart rate when compared to dipyridamole and adenosine as shown in **7B** and **C** respectively.

We tested the correlation of heart rate with MBF at rest and with stress as shown in Figures 7A, B and C. There is a good correlation between heart rate and resting MBF (R = 0. 80, 0.62 and 0.87) for regadenoson, dipyridamole and adenosine respectively. Similarly there was a modest correlation between the heart rate and stress MBF (R = 0. 49, 0.58 and 0.90) for regadenoson, dipyridamole and adenosine respectively. As shown in Figure 7A and B, compared with dipyridamole, both adenosine and regadenoson have higher MBF for similar heart rates, ie the curve is shifted upward. However with regadenoson, in addition to an upward shift the curve is also shifted right ie. a higher heart rate response (Figure 7B and C). Such an upward and rightward shift with regadenoson is noted in comparison with adenosine as well, highlighting the higher heart rate response with regadenoson. This higher heart rate response with regadenoson is not explained entirely by the decrease in systolic blood pressure. The correlation between the change in heart rate with the change in systolic BP for regadenoson is poor, R = 0.08. This suggests a direct adrenergic effect on heart rate, which is substantiated in animal studies [21].

**Figure 8:**
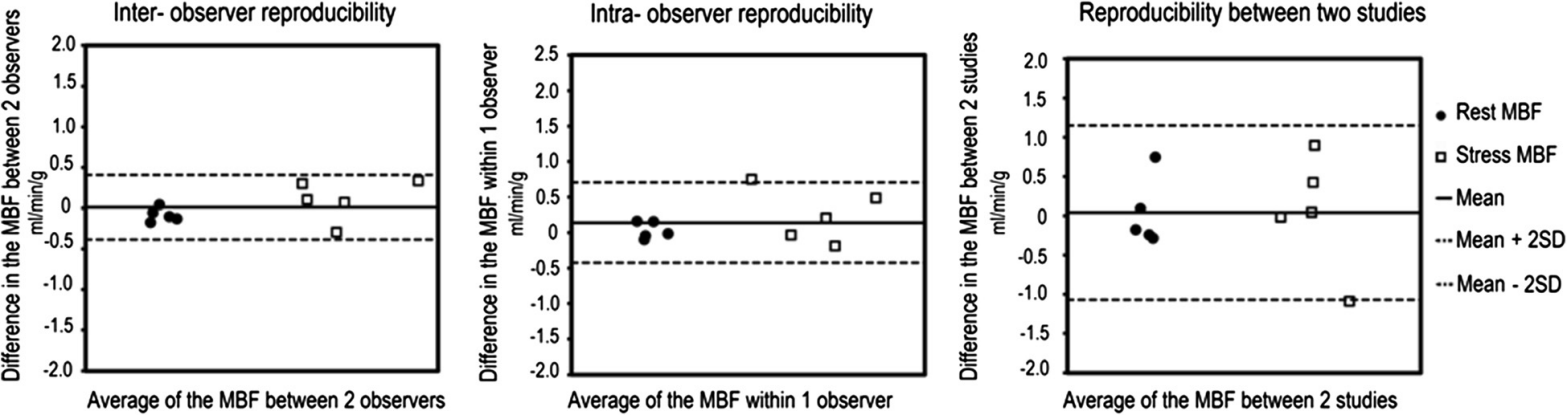
Bland-Altman plots show good inter-observer reproducibility, intra-observer reproducibility and inter-study repeatability in 5 subjects.

Measurements of resting and stress MBF in 5 subjects were analyzed by two operators to assess for both inter-observer and intra-observer reproducibility. Similarly resting and stress MBF were tested for repeatability with a second study using the same vasodilator. The Bland-Altman analysis plots showed all measurements within the 2SD range suggesting good inter- and intra-observer reproducibility (Figure 8). With the exception of one subject, rest and stress MBFs for the repeat study were within the 2SD range suggesting good day to day repeatability (Figure 8). In addition the coefficients of variation for rest MBF were 0.17, 0.15 and 0.15 and for stress MBF with regadenoson were 0.22, 0.18 and 0.19 for Observer 1, Observer 1_Repeat and Observer 2 respectively.

## Discussion

In this study of vasodilator efficacy among three FDA approved vasodilators, regadenoson had the highest stress MBF response when compared with dipyridamole and adenosine. However this is mediated primarily by a higher heart rate response with regadenoson. When adjusted for heart rate, regadenoson and adenosine have similar stress MBF and MPR, but both drugs have higher MBF and MPR than dipyridamole. To the best of our knowledge, this is the first study to directly compare the vasodilator properties in the same set of volunteers with the three commonly used vasodilators using quantitative first pass perfusion with CMR.

We expected a higher efficacy with regadenoson due to the selectivity of A2A receptors than adenosine with its nonselective action on the A1 and A3 receptors. The superior vasodilator properties of regadenoson and adenosine over dipyridamole may be explained by its direct action as an adenosine receptor agonist, in contrast to dipyridamole which causes vasodilation indirectly by increasing cellular levels of adenosine. As shown in the correlation between heart rate and MBF for each of the vasodilators, when compared with dipyridamole, adenosine has higher MBF for a given heart rate. In contrast, when compared with dipyridamole and adenosine, regadenoson has an even higher MBF and heart rate response i.e. upward and rightward shift. This increase in heart rate is not entirely explained by the changes in blood pressure and there might be a component of direct sympathetic activation causing this tachycardia. This mechanism has been substantiated in animal studies by Dhalla et al [21]. This higher heart rate response might be beneficial in the context of stress testing where increased demand can help identify ischemia. In clinical practice, these heart rate effects of regadenoson are transient and resolve by the time late gadolinium enhancement (LGE) imaging is performed (heart rate with regadenoson pre- LGE was 64 ± 12 beats per minute similar to resting heart rate, 63 ± 12 beats per minute), with no effect on subsequent image quality.

Our analysis of perfusion slices was limited to the mid myocardium. A recent study of quantitative perfusion in normal volunteers suggested that there was a modest increase in stress MBF in diastole compared to systole [22]. For quality assurance reasons, we analyzed the timing of slices within the cardiac cycle using the approach described by Feinstein et al [23]. Since only one AIF slice was acquired for each RR interval (for base, mid and apical slices) the prepulse delay was not significant. With regadenoson, which had the highest heart rate, mean of 95, the RR interval was 632 ms. The time per slice for the AIF acquisition was 60 ms, and the myocardial perfusion acquisition was 132 ms. Therefore, the AIF, base and mid slices could be acquired in 324 ms which is about 50% of the cardiac cycle, with the mean heart rate of 95 during stress. As one approaches the HR of 100, systole and diastole are similar in duration. The basal and mid slices were typically systolic, while the apical slice was diastolic (data not shown).The timing of the mid ventricular slice acquisition with all three vasodilators was in systole. Thus, the differences in stress MBF represent the differences in vasodilation between the three agents and not due to varying cardiac phase.

The impact of differences on the sensitivity and specificity of detecting coronary artery disease is limited to studies using SPECT perfusion comparing dipyridamole, adenosine and exercise stress with conflicting data [24-26]. ADVANCE-MPI (ADenoscan Versus regAdenosoN Comparative Evaluation for Myocardial Perfusion Imaging), a phase 3 multicenter international trial, showed fair concordance (chi-square coefficient of 0.63) between regadenoson and adenosine for detection of reversible perfusion defects with 99mTc-MIBI or 99mTc-Tetrofosmin [3]. ADVANCE- MPI is limited by the fair concordance even in comparisons between adenosine- adenosine scans (chi-square coefficient of 0.64) and the large number (60%) of normal scans [3,27]. Dibella et al. performed a direct comparison of myocardial perfusion reserve with adenosine and regadenoson in 8 subjects without ischemia [28]. No difference in the MPR was noted between adenosine (2.3 ± 0.9) and regadenoson (2.4 ± 0.9). Our findings are concordant with this study with similar values of MPR with adenosine and regadenoson.

While there are no differences in efficacy, regadenoson has a relative ease of administration as a rapid bolus, with a single intravenous line placement in contrast to adenosine which requires two intravenous lines and a 6 minute infusion. Regadenoson has a good safety profile in patients. In clinical studies, there was no incidence of atrioventricular block [3] and bronchospasm in patients with mild or moderate chronic obstructive pulmonary disease [29,30].

## Limitations

The study population consists predominantly of young, healthy male volunteers. We selected this population so we can test the efficacy of these three drugs. We acknowledge that response of patients to vasodilators is influenced not only by coronary artery disease but concomitant risk factors [31]. Patients might have different normal ranges of blood flow responses than young healthy volunteers. Such data will be helpful in the interpretation of quantitative perfusion.

The protocol used in this study was rest-stress imaging which is different from currently used protocols. We chose this study design due to concerns about residual vasodilation that we encountered in clinical practice and substantiated by Bhave et al [32]. This paper suggested inadequate recovery to rest blood flow with regadenoson i.e. residual vasodilation. The rest-stress study design avoids the problem of residual vasodilation and provides the best assessment of the vasodilator effects of the drugs in a study population, not expected to have LGE and heterogeneous contrast on board.

## Conclusions

Using quantitative first pass perfusion CMR in young, healthy normal volunteers, we showed that regadenoson and adenosine have similar efficacy and are a better vasodilator than dipyridamole.

## Abbreviations

MBF: Myocardial blood flow; CBF: Coronary blood flow; MPR: Myocardial perfusion reserve; ROIs: Regions of interest; CAD: Coronary artery disease.

## Competing interest

The authors declare that they have no competing interests.

## Authors’ contributions

AEA conceived the study, participated in its design and coordination, supervised perfusion quantification and image processing and helped to draft the manuscript. SV participated in the study design and conduct, analyzed the perfusion images and performed the perfusion quantification and drafted the manuscript. WPB participated in the study design and coordination and supervised the performance of the studies. CM performed the studies. LH had supervision of the perfusion quantification and helped to draft the manuscript. PK had supervision of the perfusion image processing, was responsible for sequence development and helped to draft the manuscript. SS, JW, SL supervised the performance of studies. SS, SL, JW and OB supervised the performance of the stress studies. All authors read and approved the final manuscript. 
